# Comparison of bladder ultrasonographic and rigid cystoscopic findings in patients with hematuria

**DOI:** 10.22088/cjim.10.4.417

**Published:** 2019

**Authors:** Mehrdad Mostafaloo, Naser Ghaemian, Karimollah Hajian–Tilaki, Emadoddin Moudi

**Affiliations:** 1 Student Research Committee, Babol University of Medical Sciences, Babol, Iran; 2 Clinical Research Development Center, Shahid Beheshti Hospital, Babol University of Medical Sciences, Babol, Iran; 3 Social Determinants of Health Research Center, Health Research Institute, Babol University of Medical Sciences, Babol, Iran; 4 Cancer Research Center, Health Research Institute, Babol University of Medical Sciences, Babol, Iran

**Keywords:** Sonography, Cystoscopy, Hematuria, Prostate, Bladder

## Abstract

**Background:**

Bladder cancer ranks ninth in world-wide cancer incidence and is 2.5-4 times more common in men, and painless gross hematuria is its typical clinical symptom. Cystoscopy is used to evaluate the cause of hematuria in bladder while the use and reliability of ultrasonography is questionable for this purpose. Therefore, the aim of this study was to compare abdominal ultrasonography and rigid cystoscopy in the diagnosis of bladder tumors in Babol Shahid Beheshti Hospital.

**Methods:**

In this cross-sectional study, 60 patients who were candidate for cystoscopy referred to Babol Shahid Beheshti Hospital in Iran in 2017-2018. In this study, rigid cystoscope was used to view the bladder after doing sonography. The numbers, demographic information (age, gender and residence) and clinical characteristics (indication of cystoscopy, history of chronic disease, sonographic and cystoscopic results) of patients were recorded in the checklist.

**Results:**

The mean age of patients (37 (61.7%)=males) was 58.65±14.26 (19-85) years and 48 (80%) of them were >50 years old. The sensitivity and specificity of sonography was 71.43 (95% CI: 29.4, 96.33) and 96.23% (95% CI: 87.02, 99.54) in detecting bladder tumors, respectively. The negative predictive and positive predictive values in sonography were 96.23 (95% CI: 88.76, 98.8) and 71.43% (95% CI: 37.25, 91.33) respectively.

**Conclusion:**

The results of this study indicate that since sonography has high negative predictive values in evaluating hematuria; therefore it can be replaced with rigid cystoscopy for patients with lower risk of malignancy.

The bladder is a muscular organ which stores the urine (1). Bladder cancer as the ninth common cancer in the world is 2.5-4 times more common in men (2-4). This cancer has a high recurrence rate, and the painless gross hematuria is its typical clinical presentation. Cystoscopy as a diagnostic and gold standard follow-up method, gray-scale ultrasonography as a low-cost method to check the bladder and color Doppler as a diagnostic tool for the vascularity of the lesions present in the bladder are used (1). Hematuria caused by cystitis and urinary stones is relatively prevalent and is the most common cause of referral to urology clinic. In many of these patients, especially young adults, hematuria is transient and has no specific consequences. On the other hand, there is a significant risk of malignancy caused by hematuria in older patients (>35 years), even if it is transient. However, the causes of urologic hematuria may not be diagnosed in older patients (5-10). Hematuria can be gross which is visible or microscopic which can be detected only with the urinary sediment test under the microscope. Hematuria is urologically assessed using sonography or CT scan for the upper urinary tract and cystoscopy for the lower urinary tract. 

Rigid cystoscopy is considered as an invasive intervention and has complications including dysuria, urinary retention, urosepsis and urethral injury and bleeding. Ultrasonography evaluates the lower system in addition to evaluating the upper urinary tract. This non-invasive method does not require ionizing radiation. Kumar et al. expressed that ultrasonography could be used as an initial diagnostic technique in patients with hematuria and as a technique to follow-up the patients with bladder cancer (11). The results of Gharibvand et al. have shown that ultrasonography is a cost-effective, suitable and acceptable method for diagnosis of bladder tumors (12). According to Tawfeek et al., 3-D ultrasonography due to lower costs and no radiation exposure is comparable with CT scans and MRI in virtual cystoscopy to evaluate the bladder (13). Niwa et al. suggested that there were no significant differences between ultrasonographic and cystoscopic findings in terms of tumoral characteristics of the low-risk and moderate-risk patients. Ultrasonography is also recommended in low-to moderate-risk patients (2). Stamatiou et al. reported that the sensitivity, specificity as well as positive and negative predictive values of ultrasonography were good but not as good as cystoscopy. Although the tolerability of the cystoscopy was relatively low, sonography was preferable to diagnose the causes of hematuria (14). Ahmed et al. have stated that sonography is acceptable only in the first-line diagnostic imaging in the assessment of hematuria and cannot replace cystoscopy (15). Therefore, the aim of this study was to compare abdominal ultrasonography and rigid cystoscopy in the diagnosis of bladder tumors in Babol Shahid Beheshti Hospital to introduce a better method with fewer complications and more suitable cost.

## Methods

After obtaining permission from the Ethics Committee of Babol University of Medical Sciences with the code of 12, this cross-sectional study was carried out on 60 patients who were candidates for cystoscopy and referred to Babol Shahid Beheshti Hospital considering the sensitivity and specificity of ultrasonography compared to cystoscopy (sensitivity=80% according to previous studies), error rate (0.15 d) and confidence level (0.95) in 2017-2018. 

Routine antibiotics were prescribed for all patients. The abdominal ultrasonography of the bladder was performed for the participated patients 1-7 days before the cystoscopy by the same responsible radiologist. The devices were used to do abdominal sonography of the bladder including two sonography devices, Phillips IU22 and Sonescape S50 equipped with B-mode, and tissue harmonic imaging (THI) and Doppler Color. Sonography was done using a convex probe (3-5MHz). If required, the surface probe (MHz 138) was applied for bladder dome tumors. During ultrasonography, B-mode, THI and Doppler Color were used to increase diagnostic accuracy. Color Doppler was applied to detect minimal blood flow.

Increased focal thickness of the bladder wall and any echogenic mass adhering to the bladder wall, which does not move with position change, were defined as a local tumor of the bladder wall; and each tumor based on the shape was divided into polypoid or pedunculated (height greater than width), sessile (width greater than height), infiltrative (obvious interruption of the bladder wall) and mixed. 

For each patient, the number (1, 2, 3 or >3), size (<5, 5-15, 15-25 and >25 mm) and location (Dome and Base, lateral, right or left, posterior and anterior walls and bladder neck) of the tumors were recorded in the checklist. Preparations for bladder rigid cystoscopy included no active urinary tract infections, indications for cystoscopy, sterile prep and drape, local anesthetic gel (lidocaine 10%) injection of 5-10 cc in the urethra and prophylaxis antibiotics (16). All patients were under sedation. A rigid cystoscope (Karl Storz, GmbH, Tuttlinge, Germany) with a 30-degree lens was to view the bladder base and anterolateral angle and, if necessary, a 70-degree lens was used to view the bladder neck. The number, size, location and shape of the tumors, findings of the abdominal ultrasonography and rigid cystoscopy of the bladder, demographic information (age, gender and residence) and clinical characteristics (indication of cystoscopy, history of chronic disease, ultrasonography and cystoscopy results) of patients were recorded in the checklist. In this study, all cystoscopic and sonographic procedures were performed by a urologist and a radiologist, respectively. Data were analyzed using SPSS 23 through Kappa test to compare both cystoscopic and sonographic methods (p<0.05). 

## Results

Totally, 60 patients with a cystoscopic indication, mean age of 58.65±14.26 (19-85) years and mean BMI of 27.39±4.40 (20.00-38.71 kg/m2) were examined in the running study. Table 1 presents the demographic and clinical characteristics of these patients. Among them, 48 (80%) and 37 (61.7%) patients were >50 years and males, respectively. Cystoscopic indication was microscopic hematuria, macroscopic hematuria and bladder follow-up in 40 (66.7%), 14 (23.3%) and 6 (10%) patients, respectively. Table 2 illustrates both cystoscopic and ultrasonographic results. In cystoscopy and sonography, bladder tumors were detected in 7 patients. In table 3, based on the findings of the cystoscopy, sensitivity, specificity and other characteristics of the sonography method and their 95% confidence interval are determined. The negative predictive values of ultrasound in detecting tumors was 96.23%.

**Table 1:** Patients’ demographic and clinical characteristics (n=60)

** Variable**	**Frequency (%)**
**Age**	
<50 ≥50	12(20) 48(80)
**Gender**	
Male Female	37(61.7) 23(38.3)
**Job**	
Employee Self-employment Jobless Other	19(31.7) 10(16.7) 8(13.3) 23(38.3)
**Residence**	
Urban Rural	42(70) 18(30)
**Cigarette use**	
Yes No	6(10) 54(90)
**Opium use**	
Yes No	5(8.3) 55(91.7)
**BMI (kg/m2)**	
<25 25-30 >30	20(33.3) 24(40) 16(26.7)
**Bladder history**	
Yes No	10(16.7) 50(83.3)
**lower urinary tract symptoms**	
Yes No	29 (48.3) 31 (51.7)
**Cystoscopic indication**	
Microscopic hematuria Macroscopic hematuria Follow-up	40(66.7) 14(23.3) 6(10)
**Disease history**	
Diabetes Cardiovascular No	12(20) 12(20) 36(60)

**Table 2:** Results of patients’ cystoscopy and sonography and the concordance of both methods (n=60)

**Variable **	**Cystoscopy**	**Sonography**	**Concordance**	**P-value**
**Bladder tumor**				
Yes No	7(11.7) 53(88.3)	7(11.7) 53(88.3)	5(8.3) 51(85)	<0.001
**Tumor shape**				
Polypoids Sessile Infiltrative No	5(8.3) 2(3.3) - 53(88.3)	3(5) 2(3.30 2(3.3) 53(88.3)	3(5) 191.7) - 51(85)	<0.001
**Tumor location**				
Base Bladder base Left lateral wall Right lateral wall Bladder neck	4(6.7) 2(3.3) 1(1.7) - 53(88.3)	2(3.3) 3(5) 1(1.7) 1(1.7) 53(88.3)	2(3.3) 2(3.3) 1(1.7) - 51(85)	<0.001
**Tumor length**				
<5mm mm 5-15 mm 15-25 >25 mm N	2(3.3) 2(3.3) 2(3.30 1(1.7) 53(88.3)	- 2(3.3) 2(3.3) 3(5) 53(88.3)	- 2(3.3) 2(3.3) 1(1.7) 51(85)	<0.001
**Internal flow**				
Yes No	- -	2(3.3) 58(96.8)	- -	-
**Calcification**				
Yes No	- 60(100)	- 60(100)	- 60(100)	-
**Diverticulum**				
Narrow Neck	- 60(100)	1(1.7) 59(98.3)	- 59(98.3)	-
**Trabeculation **				
Yes No	30(50) 30(50)	10(16.7) 50(83.3)	7(11.7) 27(45)	0.171
**Prostate median lobe bulging**				
Yes No	5(13.5) 32(86.5)	3(8.1) 34(91.9)	3(8.1) 32(86.5)	<0.001
**Bladder stone**				
Yes No	- 60(100)	1(1.7) 59(98.3)	1(1.7) 59(98.3)	-

The bulging of prostate median lobe was evaluated in men (n=37)

**Table 3:** Sensitivity, Specificity and other characteristics in sonography compared to cystoscopy and their 95% confidence interval

**Variable**	**Tumor**		**Trabeculation**		**Prostate median lobe bulging**	
**Cystoscopy** **Sonography**	**Positive**	**Negative**	**Positive**	**Negative**	**Positive**	**Negative**
Positive	5(8.3)	2(3.3)	7(11.7)	3(5)	3(8.1)	-
Negative	2(3.3)	51(85)	23(38.3)	27(45)	2(5.4)	32(86.5)
Sensitivity*	71.43% (29.4,96.33)		%70 (34.75, 93.3)		%80.00 28.36, 99.49)	
Specificity*	%96.23 (87.02, 99.54)		%54 (39.32, 68.19)		%91.67 (77.53, 98.25)	
Positive predictive values*	%71.43 (37.25, 91.33)		%23.33 (15.52, 33.52)		%57.14 (29.3, 81.10)	
Negative predictive values*	%96.23 (88.76, 98.8)		%90 (77.14, 96.0)		%97.06 (85.08, 99.48)	
LR+*	18.93 (4.49, 79.73)		1.52 (0.92, 2.52)		9.6 (2.98, 30.89)	
LR-*	0.30 (0.09, 0.96)		0.56 (0.21, 1.48)		0.22 (0.04, 1.26)	
Prevalence *	%11.67 (4.82, 22.57)		%16.67 (8.29, 28.52)		%12.20 (4.08, 26.2)	
Accuracy *	%93.33 (83.8, 98.15)		%56.67 (43.24, 69.41)		%90.24 (76.87, 97.28)	

* The pretenses show the 95% confidence interval for indexes of accuracy

## Discussion

In this study, cystoscopic findings were the main diagnostic basis for these patients and ultrasound findings were compared with them. None of the patients had calcification based on cystoscopy and sonography. In sonography, it was observed that only one patient had a narrow-neck diverticulum and 4 bladder stones about 5 mm in size were found in only another patient whereas these findings were not confirmed in cystoscopy. 

Ahmed et al. have stated that the sonography is an acceptable tool in the assessment of hematuria and cannot replace cystoscopy (15), which is inconsistent with the findings of the running study. The sensitivity and specificity rates of sonography were acceptable in diagnosis of bladder tumors according to Shebrya et al. (17), which is consistent with the results of the current study.

Moreover, Stamatiou et al. suggested that the cystoscopy was superior to sonography in the diagnosis of bladder tumors (14). The results of the study by Uddin et al. indicated high sensitivity and specificity of sonography (7), which is similar to the results of the current study. Sonography in Gharibvand et al.’s study was introduced as a convenient, acceptable and cost-effective method for diagnosis of bladder tumors (12). The sensitivity and specificity of sonography in their study were higher than those in the present study although both studies had almost the same sample size. They also expressed that sonography was an appropriate modality in the diagnosis of bladder tumors. In general, the results of some studies which have been conducted on the evaluation of ultrasonography compared to cystoscopy in the diagnosis of bladder tumors are in line with the results of the running study (13, 17). Though the sensitivity and specificity of sonography in several studies (11, 15) were lower than those in the current study, some studies (7, 12, 14, 17) reported that the sensitivity and specificity of this method were higher than those in the present study. 

The probability of finding malignancy in high-risk patients with hematuria is up to 40%. These patients have the history of smoking, chemical/dye exposure, recent gross hematuria or are old age (>50 years) (18, 19). Although sonograghy may be normal for these high-risk patients, it is logical to perform cystoscopy due to the high prevalence of tumors (18, 19). In this study, the number of patients and prevalence of bladder tumors are low; hence, it is recommended to do more studies with larger sample size and more specialized research community. A study with a higher sample size should be designed and implemented to assess the diagnostic value of sonography in the diagnosis of bladder tumors for patients with hematuria. A specialized study should be designed and implemented for patients with a history of bladder malignancies to compare the results of sonography and cystoscopy.

In conclusion, ultrasound had a relative high specificity but low sensitivity in the diagnosis of bladder tumors in patients with hematuria. Since sonography which is an economical and patient-accepted method has high negative predictive values in evaluating the hematuria; therefore it can be replaced with rigid cystoscopy for patients with lower risk of malignancy. 

## References

[1] Kader Abdel Hamed HA, Aly Maarouf R, Abdel Azez TT, Shoukry Sobhey S (2013). Role of multislice and virtual cystoscopy versus ultrasound and color doppler study in evaluation of urinary bladder neoplasms. Egyp J Hosp Med.

[2] Niwa N, Matsumoto K, Hayakawa N (2015). Comparison of outcomes between ultrasonography and cystoscopy in the surveillance of patients with initially diagnosed TaG1-2 bladder cancers: A matched-pair analysis. Urol Oncol.

[3] Aliramaji A, Kasaeean A, Yousefniapasha Y (2015). Age distribution types of bladder cancers and their relationship with opium consumption and smoking. Caspian J Intern Med.

[4] Shafi H, Ali Ramaji A, Akbarzadeh Pasha A (2013). A survey on 175 cases of bladder cancer in the patients who referred to the hospitals affiliated to Babol University of Medical Sciences, Iran (2001-2011). J Babol Univ Med Sci.

[5] Moudi E, Hosseini SR, Bijani A (2017). Nephrolithiasis in elderly population; effect of demographic characteristics. J Nephropathol.

[6] Moudi E, Hosseini SR, Bijani A (2016). Higher rate of microscopic hematuria in elderly patients who take regular doses of aspirin: Result from AHAP Study. Caspian J Intern Med.

[7] Uddin MM, Islam AA, Islam MS (2012). Role of flexible cystoscopy and ultrasound in the detection of recurrent bladder tumour. KYAMC J.

[8] Khadra MH, Pickard RS, Charlton M, Powell PH, Neal DE (2000). A prospective analysis of 1,930 patients with hematuria to evaluate current diagnostic practice. J Urol.

[9] Topham PS, Harper SJ, Furness PN (1994). Glomerular disease as a cause of isolated microscopic haematuria. Q J Med.

[10] Froom P, Ribak J, Benbassat J (1984). Significance of microhaematuria in young adults. Br Med J (Clin Res Ed).

[11] Kumar N, Talwar R, Nandy P (2017). Efficacy of voided urinary cytology and ultrasonography compared to cystoscopy in the detection of urinary bladder cancer. Afr J Urol.

[12] Gharibvand MM, Kazemi M, Motamedfar A, Sametzadeh M, Sahraeizadeh A (2017). The role of ultrasound in diagnosis and evaluation of bladder tumors. J Fam Med Prim Care.

[13] Tawfeek AM, Mostafa D, Mahmoud MA, Radwan A, Hamza IH (2018). The role of 3-dimensional sonography and virtual sonographic cystscopy in detection of bladder tumors. Afr J Urol.

[14] Stamatiou K, Papadoliopoulos I, Dahanis S, Zafiropoulos G, Polizois K (2009). The accuracy of ultrasonography in the diagnosis of superficial bladder s in patients presenting with hematuria. Ann Saudi Med.

[15] Ahmed FO, Hamdan HZ, Abdelgalil HB, Sharfi AA (2015). A comparison between transabdominal ultrasonographic and cystourethroscopy findings in adult Sudanese patients presenting with haematuria. Int Urol Nephrol.

[16] Messing EM, Young TB, Hunt VB, Emoto SE, Wehbie JM (1987). The significance of asymptomatic microhematuria in men 50 or more years old: findings of a home screening study using urinary dipsticks. J Urol.

[17] Shebrya NH, El Hamayed HFA, Botros SM, Shoeb MS (2014). Role of 3-dimensional ultrasonography and virtual cystoscopy in detection of bladder tumors in patients with hematuria. Egyp J Radiol Nuclear Med.

[18] Tilak P, Hydae ZZ, Moeller A, Doran TJ, Patel SG (2018). Evaluation and workup of hematuria in adults. Physician Assist Clin.

[19] Lippmann QK, Slezak JM, Menefee SA (2017). Evaluation of microscopic hematuria and risk of urologic cancer in female patients. Am J Obstet Genecol.

